# Nutraceuticals in Human Health

**DOI:** 10.3390/foods9030370

**Published:** 2020-03-23

**Authors:** Alessandra Durazzo, Massimo Lucarini, Antonello Santini

**Affiliations:** 1 CREA-Research Centre for Food and Nutrition, Via Ardeatina 546, 00178 Rome, Italy; 2 Department of Pharmacy, University of Napoli Federico II, Via D. Montesano 49, 80131 Napoli, Italy

**Keywords:** nutraceuticals, bioactive compounds, medicinal food, safety, health, regulation, clinical tests, efficacy, analysis, formulation

## Abstract

The combined and concerted action of nutrient and biologically active compounds is flagged as an indicator of a “possible beneficial role” for health. The use and applications of bioactive components cover a wide range of fields, in particular the nutraceuticals. In this context, the Special Issue entitled “Nutraceuticals in Human Health” is focused on the all aspects around the nutraceuticals, ranging from analytical aspects to clinical trials, from efficacy studies to beneficial effects on health status.

## Introduction

The combined and concerted action of nutrient components and biologically active compounds is flagged as indicator of a “possible beneficial role” for health. The use and applications of bioactive components cover a large range of fields, in particular nutraceuticals ones [1,2,3]. 

Nutraceuticals are obtained from foods of vegetal or animal origin, and the current interest and ongoing worldwide research aims to shed light and fully clarify their mechanism of action, their safety and efficacy by substantiating their role by means of clinical data [4,5]. An effort to clarify their mechanism of action will in fact open a door to a next generation of therapeutic agents that do not propose themselves as an alternative to drugs, but, instead, can be helpful to: (i) prevent a cluster of conditions that could occur together (metabolic syndrome), e.g. heart disease, stroke, and type 2 diabetes; (ii) to complement a pharmacological therapyespecially for those individuals who do not qualify for a conventional pharmacological therapy [6,7].

This Special Issue is dedicated to the role and perspectives of nutraceuticals in human health, examined from different angles, ranging from analytical aspects to clinical trials, from efficacy studies to beneficial effects on health conditions.

Concerning the study of functional ingredients and applications, Lu Martínez et al. [8] have studied and proposed the use of *Prunus* serotine defatted flour without hydrogen cyanide risk in cookies and protein concentrate in emulsion stability. Ullah et al. [9] well reviewed and summarized the biomedical properties of polysaccharides as therapeutical agents. It is worth mentioning the work of Swat et al. [10] on characterization of fulvic acid beverages available on the global market. Alternative functional ingredients obtained from waste/side products from industrial grape manufacturing, i.e., grape seeds, were investigated by Lucarini et al. [11].

Studies on evaluation of beneficial effects of nutraceuticals in vitro [12] and in vivo [13] models have been presented, in particular dietary supplementation studies in animal [14,15,16].

At the same time, specific studies on botanicals have been reported by Fredes et al. [17] and Ji et al. [18] focusing on the importance of these vegetal origin sources.

Nutraceuticals are a challenge for the future of prevention and therapy and a triggering tool in medicine area. The possibility to prevent and/or support a pharmacological therapy, which is nowadays mainly based on pharmaceuticals, can be a powerful tool to face pathological, chronic, long-term diseases in subjects who do not qualify for a pharmacological therapy. The big challenge is to improve nutraceuticals bioavailability and clear their mechanism of action adopting nanotechnologies as new delivery approach and clinical studies to assess and detail how they work in detail [19,20]. At the same time, the interest to new food sources and exploring novel nutraceuticals which beyond their nutritional value have also added value as contributing bioactive substances tailored to specific health conditions for a better results in term of efficacy and safety is stimulating interest and research worldwide for new sources and sustainable environmental friendly solutions [21,22,23].

This Special Issue end point has been to contribute to the growth of this area of research, trigger interest or research on food and add information scientifically substantiated by new data.

We would like to thank all the authors and the reviewers of the papers published in this Special Issue for their great contribution and effort. We are also grateful to the editorial board members and to the staff of the journal for their kind support during the preparation of this Special Issue.

## References

[1] Santini A., Novellino E. (2014). Nutraceuticals: Beyond the Diet before the Drugs. Curr. Bioact. Compd..

[2] Durazzo A., Lucarini M., Souto E.B., Cicala C., Caiazzo E., Izzo A.A., Novellino E., Santini A. (2019). Polyphenols: A concise overview on the chemistry, occurrence, and human health. Phytother. Res..

[3] Santini A. (2018). Nutraceuticals: Redefining a Concept. Ann. Pharmacol. Pharm..

[4] Santini A., Novellino E. (2017). To Nutraceuticals and Back: Rethinking a Concept. Foods.

[5] Daliu P., Santini A., Novellino E. (2018). A decade of nutraceutical patents: Where are we now in 2018?. Expert Opin. Ther. Pat..

[6] Santini A., Novellino E. (2018). Nutraceuticals-shedding light on the grey area between pharmaceuticals and food. Expert Rev. Clin. Pharmacol..

[7] Daliu P., Santini A., Novellino E. (2019). From pharmaceuticals to nutraceuticals: Bridging disease prevention and management. Expert Rev. Clin. Pharmacol..

[8] Lu Martínez A.A., Báez González J.G., Bautista Villarreal M., García Alanis K.G., Galindo Rodríguez S.A., García Márquez E. (2020). Studied of Defatted Flour and Protein Concentrate of *Prunus serotine* and Applications. Foods.

[9] Ullah S., Khalil A.A., Shaukat F., Song Y. (2019). Sources, Extraction and Biomedical Properties of Polysaccharides. Foods.

[10] Swat M., Rybicka I., Gliszczyńska-Świgło A. (2019). Characterization of Fulvic Acid Beverages by Mineral Profile and Antioxidant Capacity. Foods.

[11] Lucarini M., Durazzo A., Kiefer J., Santini A., Lombardi-Boccia G., Souto E.B., Romani A., Lampe A., Ferrari Nicoli S., Gabrielli P. (2020). Grape Seeds: Chromatographic Profile of Fatty Acids and Phenolic Compounds and Qualitative Analysis by FTIR-ATR Spectroscopy. Foods.

[12] Liu W.N., Shi J., Fu Y., Zhao X.H. (2019). The Stability and Activity Changes of Apigenin and Luteolin in Human Cervical Cancer Hela Cells in Response to Heat Treatment and Fe^2+^/Cu^2+^ Addition. Foods.

[13] Sayed S., Ahmed M., El-Shehawi A., Alkafafy M., Al-Otaibi S., El-Sawy H., Farouk S., El-Shazly S. (2020). Ginger Water Reduces Body Weight Gain and Improves Energy Expenditure in Rats. Foods.

[14] Omri B., Amraoui M., Tarek A., Lucarini M., Durazzo A., Cicero N., Santini A., Kamoun M. (2019). *Arthrospira Platensis* (Spirulina) Supplementation on Laying Hens’ Performance: Eggs Physical, Chemical, and Sensorial Qualities. Foods.

[15] Omri B., Alloui N., Durazzo A., Lucarini M., Aiello A., Romano R., Santini A., Abdouli H. (2019). Egg Yolk Antioxidants Profiles: Effect of Diet Supplementation with Linseeds and Tomato-Red Pepper Mixture before and after Storage. Foods.

[16] Omri B., Larbi Manel B., Jihed Z., Durazzo A., Lucarini M., Romano R., Santini A., Abdouli H. (2019). Effect of a Combination of Fenugreek Seeds, Linseeds, Garlic and Copper Sulfate on Laying Hens Performances, Egg Physical and Chemical Qualities. Foods.

[17] Fredes C., Parada A., Salinas J., Robert P. (2020). Phytochemicals and Traditional Use of Two Southernmost Chilean Berry Fruits: Murta (*Ugni molinae* Turcz) and Calafate (*Berberis buxifolia* Lam.). Foods.

[18] Ji M.Y., Bo A., Yang M., Xu J.F., Jiang L.L., Zhou B.-C., Li M.H. (2020). The Pharmacological Effects and Health Benefits of Platycodon grandifloras-A Medicine Food Homology Species. Foods.

[19] Souto E.B., Silva G.F., Dias-Ferreira J., Zielinska A., Ventura F., Durazzo A., Lucarini M., Novellino E., Santini A. (2020). Nanopharmaceutics: Part I-Clinical Trials Legislation and Good Manufacturing Practices (GMP) of Nanotherapeutics in the EU. Pharmaceutics.

[20] Souto E.B., Silva G.F., Dias-Ferreira J., Zielinska A., Ventura F., Durazzo A., Lucarini M., Novellino E., Santini A. (2020). Nanopharmaceutics: Part II-Production Scales and Clinically Compliant Production Methods. Nanomaterials.

[21] Campos J.R., Severino P., Ferreira C.S., Zielinska A., Santini A., Souto S.B., Souto E.B. (2019). Linseed Essential Oil—Source of Lipids as Active Ingredients for Pharmaceuticals and Nutraceuticals. Curr. Med. Chem..

[22] Salehi B., Venditti A., Sharifi-Rad M., Kregiel D., Sharifi-Rad J., Durazzo A., Lucarini M., Santini A., Souto E.B., Novellino E. (2019). The Therapeutic Potential of Apigenin. Int. J. Mol. Sci..

[23] Lucarini M., Durazzo A., Romani A., Campo M., Lombardi-Boccia G., Cecchini F. (2018). Bio-Based Compounds from Grape Seeds: A Biorefinery Approach. Molecules.

